# Neuroendocrine Tumor Arising within Mature Cystic Teratoma of the Pancreas: Literature Review and Case Report

**DOI:** 10.3390/curroncol29070374

**Published:** 2022-07-06

**Authors:** Mihajlo Djokic, Benjamin Hadzialjevic, Branislava Rankovic, Rok Dezman, Ales Tomazic

**Affiliations:** 1 Department of Abdominal Surgery, Ljubljana University Medical Center, Zaloska Cesta 7, 1000 Ljubljana, Slovenia; mihajlo.djokic@kclj.si (M.D.); benjamin.hadzialjevic@kclj.si (B.H.); 2 Department of Surgery, Faculty of Medicine, University of Ljubljana, Zaloska Cesta 7, 1000 Ljubljana, Slovenia; 3 Institute of Pathology, Faculty of Medicine, University of Ljubljana, Korytkova Ulica 2, 1000 Ljubljana, Slovenia; branislava.rankovic@mf.uni-lj.si; 4 Department of Radiology, Ljubljana University Medical Center, Zaloska Cesta 7, 1000 Ljubljana, Slovenia; rok.dezman@kclj.si

**Keywords:** germ cell tumor, mature teratoma, extragonadal teratoma, neuroendocrine tumor, cystic tumor, pancreas, surgery

## Abstract

Cystic teratomas are germ cell tumors most commonly found in the ovaries and testes. The pancreas, however, is very rare as a site of occurrence. Moreover, only two cases of cystic teratoma with concomitant neuroendocrine tumor have been reported to date. We report the case of a 33-year-old female who presented with abdominal pain. Computed tomography and magnetic resonance imaging of the upper abdomen revealed an 85 mm cystic tumor in the head of the pancreas. Cystic teratoma and mucinous cystadenoma were suggested as differential diagnoses. Cytopathologic analysis of endoscopic ultrasound-guided fine needle aspiration was consistent with mucinous cystadenoma. Therefore, the patient underwent surgical resection. Histologic analysis revealed a mature cystic teratoma of the pancreas with a concomitant neuroendocrine tumor. The patient is in great condition at 8 months follow-up. Cystic teratoma of the pancreas with a concomitant neuroendocrine tumor is an extremely rare condition. Surgical resection remains the mainstay of treatment as it provides a definitive diagnosis and no recurrences have been reported to date.

## 1. Introduction

Teratoma is a germ cell tumor composed of tissues derived from at least two embryonic layers (ectoderm, endoderm, or mesoderm). Teratomas arise most commonly in the ovaries and testes, but can also develop from germ cells that have arrested in their migration anywhere along the midline. Therefore, extragonadal teratomas are commonly found in the cranium, mediastinum, retroperitoneum, and pelvis (especially sacrococcygeal region) [1,2]. The pancreas, though, is very rare as a site of occurrence. Since the first report in 1918 by Kerr et al., slightly more than 50 cases of pancreatic teratomas have been described to date [3,4]. In addition, to the best of our knowledge, only two cases of pancreatic teratoma with concomitant neuroendocrine tumor have been reported so far [5,6].

We present a case of mature cystic teratoma with concomitant neuroendocrine tumor in the head of the pancreas of a 33-year-old woman.

## 2. Case Presentation

**Figure 1:**
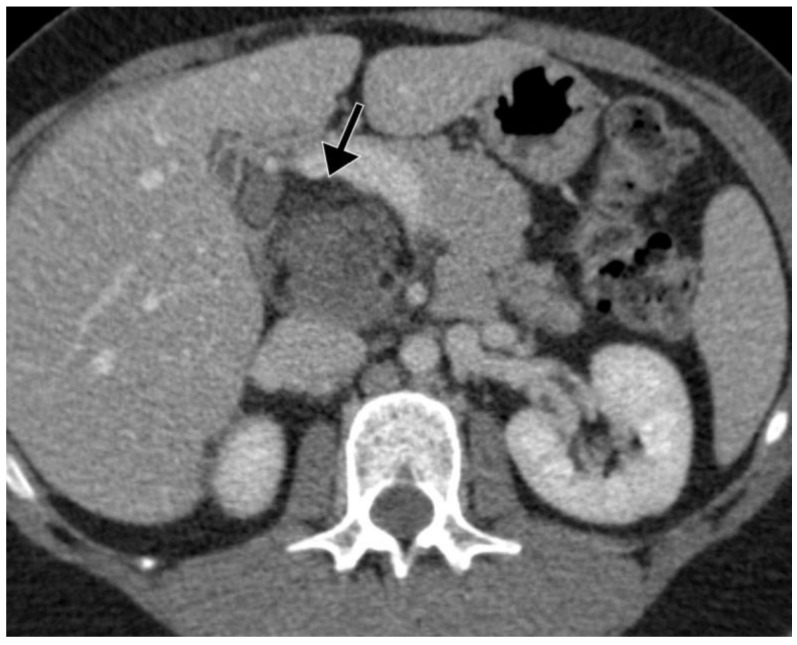
Contrast enhanced CT of the abdomen demonstrates a well demarcated retroperitoneal tumor posterior to the pancreatic head. Notice the presence of the macroscopic fat (black arrow), a characteristic finding consistent with teratoma.

**Figure 2:**
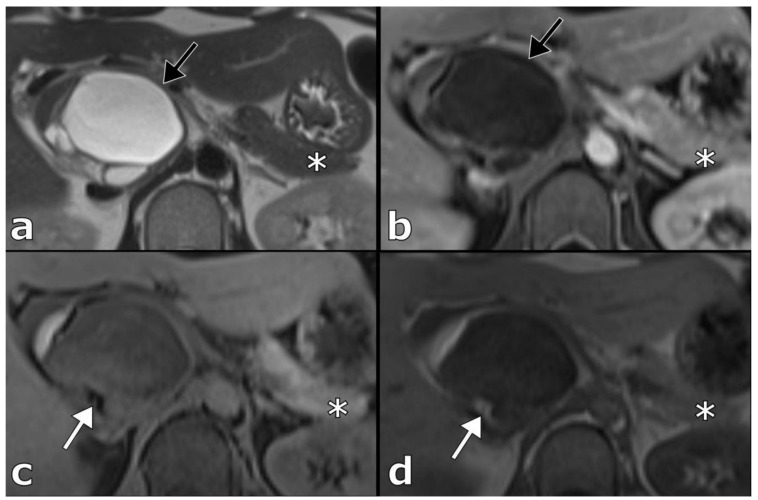
Contrast enhanced MRI of the abdomen. T2 weighted images (**a**) demonstrate the predominantly cystic component of the tumor with thin wall (black arrow), that has no apparent enhancement on post-contrast imaging (**b**). T1-weighted imaging (**c**) depicts an area of the high-signal intensity in the tumor (white arrow) with the corresponding signal-drop on the T1 fat saturated sequence (**d**), indicating the presence of macroscopic fat tissue in the tumor. Notice the absence of the dilation of the pancreatic duct (asterisk, all sequences).

A 33-year-old Caucasian female with no chronic medical conditions consulted her primary care physician for upper abdominal pain. Initial routine laboratory tests were within normal ranges. Abdominal ultrasound (US) revealed a 42 mm, round, inhomogeneous mass in the region of the pancreatic head. Contrast-enhanced computed tomography (CT) of the abdomen showed an 83 × 46 × 46 mm tumor located retroperitoneally between the head of the pancreas and the inferior vena cava (IVC). This well demarcated tumor had a polycystic component with thin septations and macroscopic fat inclusions (Figure 1). Pancreatic duct was not dilated and no other abnormalities were identified elsewhere in the abdomen. Further contrast enhanced magnetic resonance imaging (MRI) of the upper abdomen confirmed an 85 × 56 × 53 mm macrocystic tumor mass in the head of the pancreas. There was no invasion of surrounding structures (Figure 2).

The patient was referred to our department for further evaluation of the above-mentioned cystic tumor mass. She reported discomfort in the upper abdomen and denied weight loss, pruritus, jaundice, or changes in her appetite. No mass or other signs were detected during the physical examination. Laboratory tests, including liver and pancreatic function tests, were within normal ranges. Additional tumor marker testing revealed an elevated cancer antigen 125 (CA 125) level of 94.5 kU/L (normal up to 35 kU/L), slightly elevated cancer antigen 19-9 (CA 19-9) level of 42.4 kU/L (normal up to 37), and carcinoembryonic antigen (CEA) level of 6.2 kU/L (normal up to 4.2 kU/L), while alpha-fetoprotein (AFP) levels were within normal limits.

**Figure 3:**
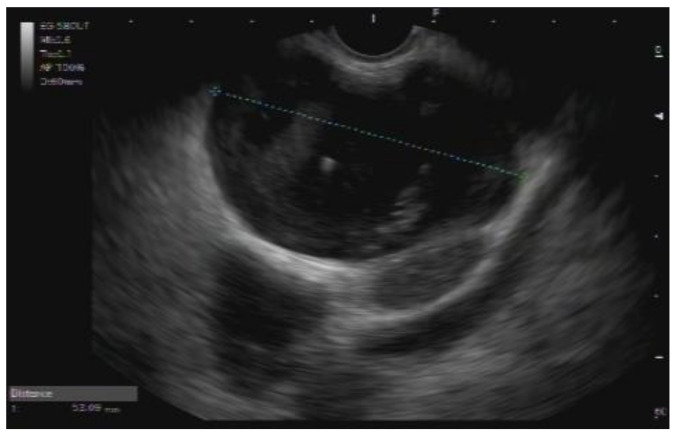
EUS demonstrates a 52 mm, mostly anechoic cystic lesion between the posterior gastric wall and the head/body of the pancreas.

Based on the CT and MRI findings, retroperitoneal/pancreatic teratoma and mucinous cystadenoma were suggested as differential diagnoses. However, because imaging studies were inconclusive, further evaluation with endoscopic US (EUS)-guided fine-needle aspiration (FNA) was recommended. Thus, EUS demonstrated a 52 mm, mostly anechoic cystic lesion with some hyperechoic areas located between the posterior gastric wall and the head/body of the pancreas (Figure 3). Pancreatic parenchyma and pancreatic duct were both unremarkable. FNA of the lesion was performed and a viscous, colorless cystic fluid was obtained. Cytopathologic analysis revealed a dense mucoid substance with glandular cells with low-grade atypia. Alcian blue staining was positive. Scattered macrophages, siderophages, neutrophils, and lymphocytes were also observed. Based on these features, a diagnosis of mucinous cystic neoplasm was suggested.

Considering the preoperative diagnosis of mucinous cystadenoma, a surgical removal of the lesion was recommended. Thus, laparotomy was performed in the usual manner. Abdominal exploration revealed a large cystic tumor in the head of the pancreas. The tumor was not in direct contact with any other organs or vessels. Therefore, a pylorus preserving pancreaticoduodenectomy (PPPD) was performed. Postoperatively the patient developed pneumonia which was treated with antibiotics. She also had percutaneous drainage of the intraabdominal collection. However, amylase and lipase levels in the drains remained negative. The patient was discharged on the 12th postoperative day.

**Figure 4:**
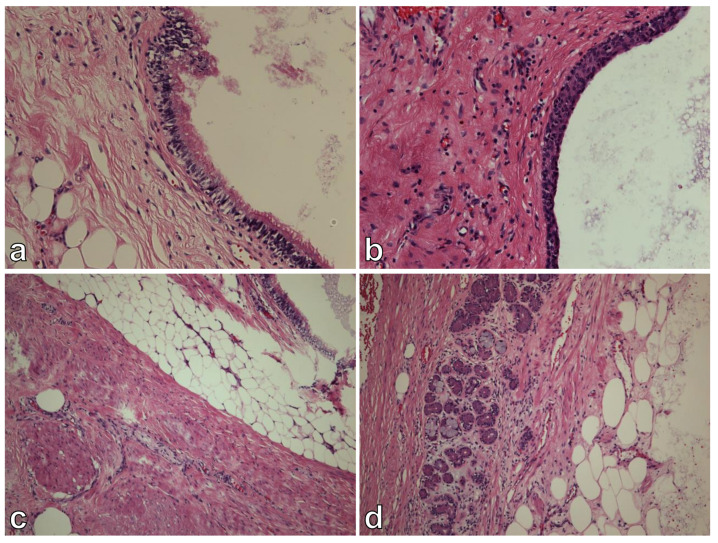
Histologic analysis. Cystic tumor with respiratory (**a**) and squamous cell (**b**) epithelium lining. Fibro adipose tissue, muscle cells, and salivary glands were also observed (**c**,**d**).

**Figure 5:**
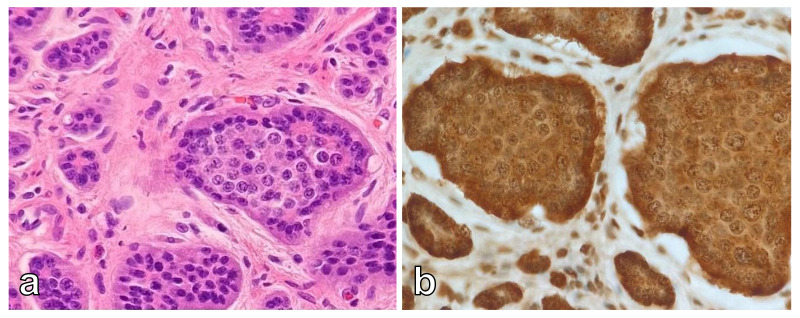
Histologic analysis and immunohistochemistry. Well differentiated neuroendocrine tumor with organoid architecture (**a**). Chromogranin diffuse positive reaction (**b**).

On gross examination, the tumor was multiloculated, cystic, and confined to the pancreas. For histologic examination, in order to exclude a possible immature component, the whole tumor was sampled. Histologic analysis revealed a mixture of benign mature tissues representing all three embryonic layers—ectodermal, mesodermal, and endodermal (respiratory epithelium, squamous epithelium, salivary glands, fibro adipose tissue). Throughout the tumor we have not observed any solid areas, areas with primitive neuroectodermal components (spindle cells, rosettes, pseudorosettes, primitive tubules), or areas with hemorrhages or necrosis that could be related with malignancy. Therefore, the findings were consistent with mature cystic teratoma (Figure 4). In addition, a small, 6 mm tumor was observed, consisting of monomorphic round and oval cells with salt and pepper chromatin. By immunohistochemistry, cells were positive for neuroendocrine markers synaptophysin and chromogranin (Figure 5). Morphological and immunohistochemical features were consistent with neuroendocrine tumor. The Ki67 proliferation index was less than 2%. No lymphovascular or perineural invasion was present.

The patient is in great condition at 8 months follow-up with no radiological or clinical evidence of recurrence. In addition, tumor markers have decreased within normal limits.

## 3. Discussion

Teratomas are germ cell tumors that arise from tissues of at least two germ cell layers. Furthermore, depending on the degree of differentiation of their components, they can be subdivided into mature (cystic or solid), immature (malignant), and monodermal or highly specialized. Of these three groups, mature cystic teratomas are the most common. Mature cystic teratomas are often referred to as dermoid cysts due to their preponderance toward ectodermal differentiation [7]. They are most commonly found in the gonads, but can arise in almost any structure along the midline of the body, with the pancreas among the rarest sites of occurrence [8,9]. Moreover, pancreatic neuroendocrine tumors are also rare heterogenous pancreatic diseases, accounting for less than 3% of all pancreatic neoplasms [10,11]. Consequently, to the best of our knowledge, only two cases of pancreatic teratoma with concomitant neuroendocrine tumor have been reported so far [5,6].

The etiopathogenesis of extragonadal germ cell tumors is still poorly understood. The most established hypothesis is that extragonadal germ cell tumors originate from primordial germ cells that failed to leave the nerve branches at the gonadal site and therefore continued their migration along the midline of the body. Consequently, they could end up at other, more distant sites and, unless eliminated by apoptosis, they could give rise to extragonadal germ cell tumors [12]. On the other hand, it has also been suggested that extragonadal germ cell tumors in adults represent distant metastases of an undetected or occult germ cell tumor of the gonads [2]. In our case, we were unable to elucidate the precise pathogenesis of the mature cystic teratoma; however, we were at least partly able to refuse the latter hypothesis of metastatic origin because no tumor was visible in our patient’s ovaries. Malignant transformation is a rare complication of mature cystic teratoma. It has been reported in two large case series of mature cystic teratomas of the ovary, as they are a common pathology of the ovary. The incidence of malignant transformation ranged from 2.4% to 3.5%. Squamous cell carcinoma was the most common neoplasm within mature cystic teratoma, while adenocarcinoma or malignant teratoma were extremely rare [13,14]. However, such cases have not been reported in mature cystic teratomas of the pancreas.

Cystic teratoma of the pancreas has been reported in both children and adults, with the youngest case described in a 4-month-old girl and the oldest case in 74-year-old man [15,16]. It is usually observed in the second to third decade of life. As noted, cystic teratoma affects both sexes, however it has a slight male gender preponderance [17]. Although cystic teratomas have been found in all parts of the pancreas, they occur more frequently in the body and head, followed by the tail of the pancreas. The size of the tumor ranged from 2.2 cm to 25 cm [18]. Thus, our case is mostly consistent with the commonly presented features of previous reports.

Most patients had nonspecific symptoms at the time of diagnosis. As in our patient, abdominal pain was the most common symptom, while few patients presented with back pain, jaundice, and vomiting. The latter was more prevalent in pediatric patients [9,19]. Asymptomatic cystic teratomas of the pancreas have been most frequently detected in recent years, probably due to the increasing use of imaging modalities [20].

Although serum tumor markers can help to distinguish benign lesions from malignant ones, this could not be attributed for cystic teratomas of the pancreas, as tumor marker values have been reported in only a few cases, most of which remained negative. Nevertheless, slightly elevated levels of CA 19-9, CEA, and CA 125 have been reported, as in our case [7,9,21]. In the two cases in which cystic teratoma and neuroendocrine tumor occurred simultaneously, no values for serum tumor markers were reported [5,6]. Nevertheless, serum tumor markers are neither disease nor organ specific and several other factors may influence their levels [22].

The development and increased use of imaging techniques has resulted in a growing number of pancreatic cystic lesions being detected recently. This also applies for pancreatic cystic teratomas, as approximately half of all reports of pancreatic teratomas have been described only in the last 10 years. Consequently, it is paramount and sometimes challenging to differentiate benign cysts (pseudocyst, serous cystadenoma) from potentially malignant cysts (intraductal papillary mucinous neoplasm, mucinous cystadenoma), while uncommon pancreatic lesions present even a tougher challenge to many radiologists [23,24].

The radiological appearance of cystic teratomas of the pancreas varies depending on the proportion of tissue components presented within the cyst [25]. Thus, their appearance on abdominal US and EUS varies from anechoic to hyperechoic masses. Additionally, hyperechoic mural nodules may been seen arising from the cyst wall [26,27,28]. CT reports of cystic pancreatic teratomas include round, thin-walled, well circumscribed lesions, either unilocular or with septa, whereas characteristic fat-fluid levels are rarely observed. In addition, macroscopic fat inclusion within the cyst, also a characteristic finding in cystic teratoma, may rarely be seen on CT, as in our case [24,29]. MRI scans typically show fatty content as a signal with high intensity on T1-weighted images and variable intensity on T2 weighted images [27].

Recently, several studies have been published on the management of pancreatic cystic neoplasms. In general, patients with unusual cystic tumors of pancreas larger than 30 mm should undergo EUS and preferably also EUS-FNA to differentiate mucinous from non-mucinous cystic neoplasms [30]. This was also the case in our patient, and since cytopathologic examination suggested a mucinous cystadenoma, she underwent surgical resection. Although EUS-FNA is usually helpful in establishing the correct diagnosis of pancreatic cystic neoplasms, dissociation with the final surgical diagnosis may occur, as in our case. Cytology from EUS-FNA aspirates has high specificity (88–93%), but relatively low sensitivity (54–63%), resulting in low diagnostic accuracy [30,31]. In addition, mucin stains (e.g., alcian blue) may be interpreted as false positives due to possible gastrointestinal contamination of wisps of mucin into the sample during FNA [32]. Thus, contamination could also be the reason for the false positive alcian blue test in our case. Nevertheless, two successful preoperative diagnoses of cystic teratomas of the pancreas by EUS-FNA have been described [5,25].

Surgical resection remains the mainstay in the treatment of cystic teratomas of pancreas, as it consequently provides a definitive diagnosis and no recurrences of the disease have been reported to date [6]. Hypothetically, smaller mature cystic teratomas could be managed under observation knowing the final diagnosis, due to their benign nature. This is also supported by a case report in which the patient was initially diagnosed with a pancreatic pseudocyst by abdominal US examination, which eventually turned out to be a pancreatic teratoma 30 years later according to histologic analysis of the surgical specimen. During this time, the patient had multiple drainage procedures and her quality of life was significantly impaired [18]. Nevertheless, since it is difficult to exclude malignant potential preoperatively and because they eventually begin to cause symptoms, cystic teratomas of the pancreas should be treated with surgical resection. In addition, as in our case, a neuroendocrine tumor may arise within a pancreatic teratoma [5,6]. Drainage procedures were performed in the earlier reports of pancreatic teratomas and they should be avoided because of the higher likelihood of persistent fistula formation [3,9,33].

The main characteristics of our patient and the other two patients with concomitant mature cystic teratoma of the pancreas and neuroendocrine tumor are summarized in Table 1. Interestingly, several similarities can be observed when compared with our patient. They were all symptomatic and in their thirties. They were treated with surgical resection according to the location of the tumor. Moreover, the size of the cystic teratoma was more than 7 cm, whereas the size of the neuroendocrine tumor was less than 1 cm in all three patients. Unfortunately, no follow-up was reported in either of the first two cases [5,6].

## 4. Conclusions

Cystic teratoma of the pancreas with concomitant neuroendocrine tumor is an extremely rare condition with unclear pathogenesis. However, it should be considered in young patients with a large symptomatic cystic lesion of the pancreas associated with elevation of tumor markers. Surgical resection has been demonstrated to be both diagnostic and curative in the management since it provides final diagnosis and no recurrences have been reported to date.

## Figures and Tables

**Table 1:** Characteristics of patients with concomitant mature cystic teratoma of the pancreas and neuroendocrine tumor.

Author, Year	Age	Sex	Symptoms	Location	Treatment	Cystic Teratoma Size	NET Size	NET Proliferation Index
Mateos et al. [5], 2010	39	F	UA pain	Body/tail	Distal pancreatectomy and splenectomy	85 × 76 mm	8 mm	NP
Rojas et al. [6], 2021	35	M	LUQ pain	Head	Cephalic duodenopancreatectomy	71 × 53 mm	3 mm	<1%
Djokic et al., 2022 (current paper)	33	F	UA pain	Head	PPPD	85 × 56 mm	6 mm	<2%

*LUQ*, left upper quadrant; *NET*, neuroendocrine tumor; *NP*, not provided; *PPPD*, pylorus preserving pancreaticoduodenectomy; *UA*, upper abdominal.

## Data Availability

The data presented in this study are available on request from the corresponding author.
